# Non-variable RNA deletion using the CRISPR-Cas9 technique demonstrated improved outcomes in human intestine single-cell RNA sequencing data, even at half sequencing depths

**DOI:** 10.1186/s44342-025-00043-6

**Published:** 2025-05-20

**Authors:** Dong Jun Kim, Christine Suh Yun Joh, So Young Jeong, Yong Jun Kim, Seong Joon Koh, Hyun Je Kim

**Affiliations:** 1 Department of Biomedical Sciences, Seoul National University Graduate School, Seoul, 03080 Republic of Korea; 2 Department of Microbiology and Immunology, Seoul National University College of Medicine, Seoul, 03080 Republic of Korea; 3 Cancer Research Institute, Seoul National University College of Medicine, Seoul, 03080 Republic of Korea; 4 Genomic Medicine Institute, Seoul National University College of Medicine, Seoul, 03080 Republic of Korea; 5 Interdisciplinary Program in Artificial Intelligence (IPAI), Seoul National University, Seoul, 08826 Republic of Korea; 6 Department of Internal Medicine and Liver Research Institute, Medical Research Center, Seoul National University College of Medicine, Seoul, 03080 Republic of Korea

**Keywords:** CRISPR-Cas9, Non-variable RNA, Single cell RNA sequencing

## Abstract

In single-cell RNA sequencing (scRNA-seq) data, issues related to the high expression of non-variable RNAs often arise due to organ traits or sample quality. Computational methods, such as SoupX (Young (Gigascience 9:giaa151, 2020)), have been used to solve this problem but it may remove biologically relevant data. This study presents a clustered regularly interspaced short palindromic repeats (CRISPR)-Cas9-based method that selectively removes non-variable RNAs. We applied this approach to scRNA-seq data from human intestinal tissues of 17 patients. By targeting non-variable genes, including ribosomal and mitochondrial RNAs, CRISPR-Cas9 treatment effectively reduced their expression, outperforming computational methods in both the number and extent of gene removal. The CRISPR-Cas9 treated samples, sequenced at half the depth compared to untreated samples, maintained comparable sequencing quality, and saturation, demonstrating that this approach can reduce sequencing costs while preserving data quality. Cell type composition and gene expression patterns remained consistent between treated and original datasets, with no unintended gene deletions. Overall, our findings suggest that the CRISPR-Cas9-based method offers a cost-effective solution for improving scRNA-seq data quality, particularly for tissues with high levels of non-variable RNAs, without compromising biological integrity.

**Supplementary Information:**

The online version contains supplementary material available at 10.1186/s44342-025-00043-6.

## Introduction

The cost of whole genome sequencing (WGS) has dramatically decreased since 2008 [1]; however, the cost of single-cell RNA sequencing (scRNA-seq) data remains relatively expensive due to its large data size. The recommended sequencing parameters from 10X Genomics are shown in Table 1 [2]. According to these guidelines, the minimum sequencing depth is 20,000 read pairs/cell with 200 sequencing cycles. For a typical input of 20,000 cells (a commonly used sample size) [2], the total data size would be approximately 80 GB. The sequencing depth will be more higher according to sample trait and size. So the size of scRNA-seq data is basically huge, it makes high cost burden.

**Table 1:** Single-cell RNA data library sequencing depth and run parameter

Sequencing depth	Minimum 20,000 read pairs per cell
Sequencing type	Paired end, dual indexing
Sequencing read	Index 1: 28 cycles, 90 cycles Index 2: 10 cycles

Non-variable RNAs refer to RNAs that encode genes ubiquitously expressed across all cells, such as mitochondria related genes or ribosomal genes. In scRNA-seq data, we often encounter issues related to non-variable RNAs. Their high expression levels make it difficult to detect lower-abundance genes, as they consume a substantial portion of the limited sequencing capacity. Scientifically, these RNAs interfere with data interpretation and even introduce distortions. For example, they can disrupt network or interaction analyses and cause the expression levels of other genes to be underestimated. Various computational method (in silico) are developed to solve this problem. One of the widely used method is SoupX which removes RNAs with high contamination fraction [3]. However, its performance is limited and carries the risk of removing biologically relevant data.

CRISPR-Cas9, a gene-editing technology known for its high specificity, is widely used in biological research [4]. Notably, in scRNA-seq, CRISPR-Cas9 technology can remove the targeted genes before PCR amplification, preventing these genes from being amplified. This ensures their successful exclusion from sequencing data. This approach is particularly advantageous for studies involving intestinal tissues, which contain high levels of enzyme such as DNase and RNase [5], leading to extensive RNA degradation. During experiment, RNA fragments generated by enzymatic degradation often enter single-cell droplets, increasing background noise in intestinal scRNA-seq data [6]. Although in silico methods are commonly applied to intestinal dataset, they can be inefficient and risk removing unintended data.

Previous studies have individually reported the gene removal efficiency of CRISPR-Cas9 and computational methods. However, a direct comparison between the two methods and a detailed analysis of their respective characteristics have not yet been conducted. This study aims to fill this gap. Furthermore, previous reports on CRISPR-Cas9 system used in our study did not examine off-target effects or conduct more comprehensive downstream analyses. In this study, we extended the evaluation to global gene expression to determine whether CRISPR-Cas9 selectively removes target genes without compromising the integrity of the original data.

## Materials and methods

### Cohort and sample collection

A total of 17 patients were diagnosed with IBD at Seoul National University Hospital were included in this study. Intestinal tissue samples were collected during endoscopy, comprising 15 inflamed sites and 16 non-inflamed sites. In short, the intestine tissues were enzymatically digested using collagenase (Liberase TL, Merck) and an RNase inhibitor (Roche) to isolate cells. The isolated cells were frozen in cryopreservation medium (CELLBANKER, Zenogen Pharma) and stored in − 80 °C deep freezer for 24 h and transferred to liquid nitrogen for long-term storage.

After thawing, cryopreserved cells were washed twice in a 37 °C Roswell Park Memorial Institute (RPMI) 1640 Media containing 10% FBS (Sigma). Cells were resuspended in 500 μL 10% FBS in RPMI1640 and counted using LUNA-FL (Logos Biosystem). Cell viability was also counted using LUNA-FL.

### Single cell preparation and sequencing

Isolated cells were counted to contain 10,000 cells for each sample, aiming to yield 5000 cells per individual. In general, cells from four samples were pooled to form a single Gel-in-emulsion (GEM), resulting in a total of eight GEMs. (GEM2 contained cells from only three samples due to the poor condition of one sample). The pooled GEMs contained a mixture of both sexes and ages. Single cell GEM generation and library construction were performed using the Chromium Next GEM Single cell 5′ HT v2 platform. (10X Genomics, document CG000423). GEM and cDNA were generated using the Chromium Next GEM Single Cell 5′ HT Kit v2, (10X Genomics, PN- 1000356). And GEX libraries were constructed using Library Construction Kit (PN- 1000352). During the library construction process, CRISPR-Cas9 (JUMPCODE GENOMICS, DepleteX, KIT1018) was used to selectively eliminate non-variable genes. The list of guide RNAs is provided in the Supplementary Table S1. The quality of GEX libraries were assessed using 4200 Tapestation system (Agilent). The constructed GEX libraries were sequenced on Illumina NovaSeq 6000. CRISPR-Cas9 treated libraries were sequenced at 50,000 reads per cell, while the untreated (original) were sequenced at 25,000 reads per cell. The number of sequencing cycles were 200.

### CRISPR-Cas9-based cDNA deletion

Immediately after the library ligation step, a ribonucleoprotein (RNP) Complex was assembled using guide RNA, Cas9 buffer, RNase inhibitor and Cas9 enzyme from the DepleteX kit (JUMPCODE GENOMICS, KIT1018) following the manufacturer's protocol. The RNP complex was then add to adaptor-ligated cDNA library and incubated at 42℃ for 1 h. After incubation, the cDNA was purified using AMPure XP beads (Beckman Coulter, A63881) following the manufacturer’s protocol. Gene expression library construction was resumed from sample index PCR step.

### Single cell RNA sequencing data preprocessing and QC

Cell Ranger version 7.0.1 was used to align the FASTQ files to the GRCh38 reference genome (10X Genomics). Patient genomic DNA from blood samples was used to identify single nucleotide polymorphism (SNP), which were then utilized to distinguish individual patient information. The processed matrix was analyzed with Seurat (version 4.4.0). SoupX (version 1.6.2) was used to remove ambient RNA contamination, with the contamination fraction automatically estimated using autoEstCont function. With Seurat, basic quality control, normalization, scaling, dimension reduction, clustering and UMAP visualization were done. Each sample data underwent filtration based on criteria as total UMI counts > 500 and mitochondrial gene percentage < 25%. After the filtering, a total number of 342,795 cells were retained (183,061 cells from the original data and 159,734 cells from the CRISPR-Cas9-treated data). In SoupX data, 187,559 cells were recovered. Normalization was performed using logarithm method, and the top 2000 variable genes were identified for dimensionality reduction using Principal component analysis (PCA). scDblFinder was used to compute and remove doublet based on probability estimation.

### Integration, Pathway enrichment, Differential expression analysis (DEG), Gene scoring, and Down sampling

To correct for batch effects, Harmony integration and CCA integration were applied. The appropriate dimension was calculated using mathematical method. Differently expressed genes were identified using FindAllMarkers function in Seurat. Genes were limited with a log-fold change > 0.25 and minimum percentage (min.pct) > 0.25. The annotation of cell clusters was done manually, based on a well-established reference study. Gene Ontology (GO) analysis was conducted to infer pathway enrichment for each cell population, using the top 200 genes per cluster. To assess non-variable RNA expression, a non-variable gene score was generated using the AddModuleScore function in Seurat, incorporating approximately 100 ribosomal and mitochondrial genes. Down sampling was done by Seurat package. Five hundred cells were sampled randomly from each GEM.

### Statistics

Wilcoxon rank sum test for detecting differentially expressed genes was performed via FindAllMakers and FindMarkers functions. GraphPad Prism version 8.4.2 was used and paired T test was used to analyze *p*-value in paired groups.

## Results

### CRISPR-Cas9 based method removes mitochondrial RNA and ribosomal RNA more accurately

**Fig. 1:**
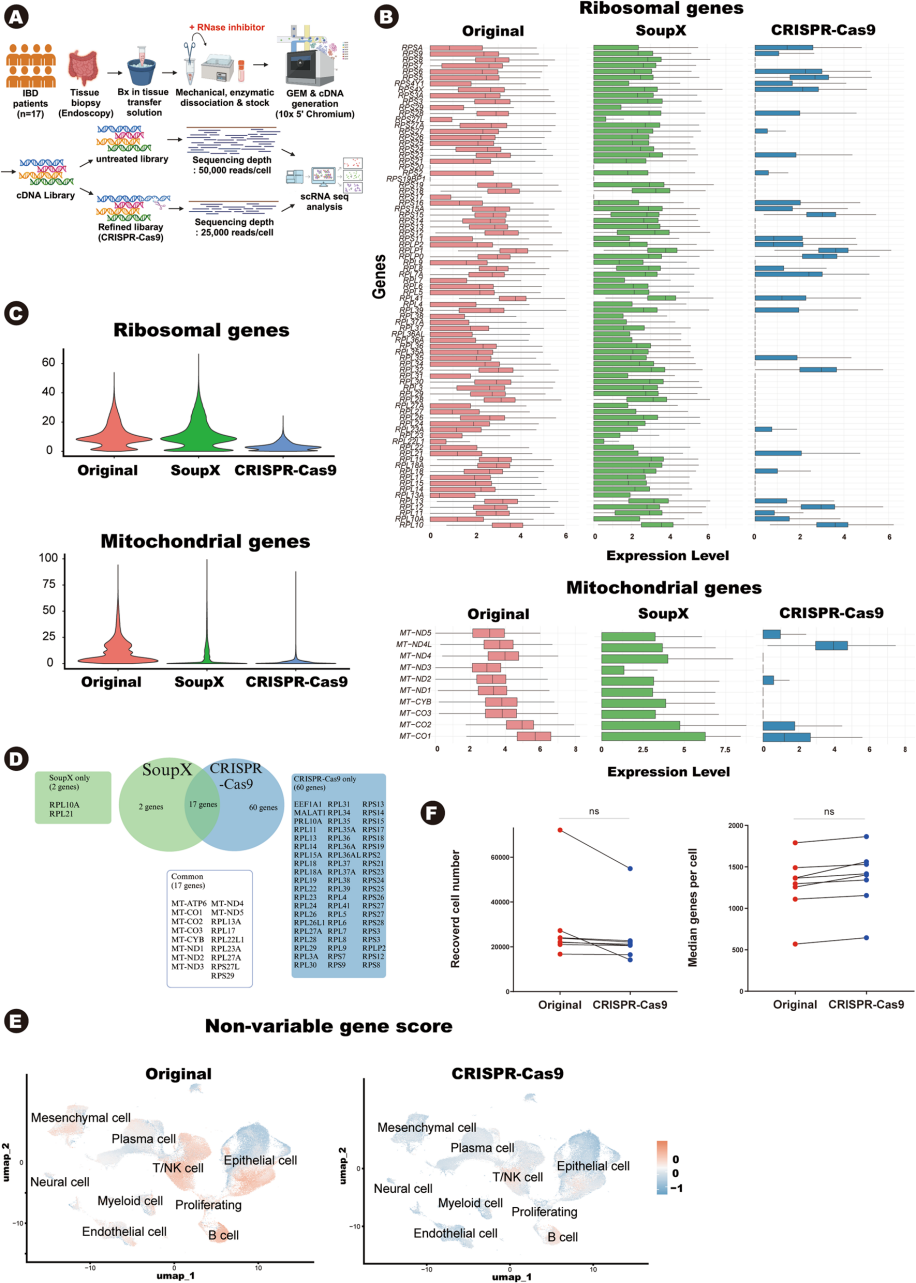
**A** Overview of human intestinal tissue sampling and scRNA-seq workflow. scRNA-seq was performed on intestinal tissue from IBD patients. During the GEX library construction process, a subset of libraries was treated with CRISPR-Cas9 targeting non-variable genes. Libraries treated with CRISPR-Cas9 were sequenced at half of the sequencing depth. **B** The boxplots display the expression levels of ribosomal genes (top) and mitochondrial genes (bottom) across three groups : original data (left), computationally processed data using SoupX (middle), and CRISPR-Cas9 treated data (right). **C **The violin plot, illustrate the expression levels of ribosomal genes (top) and mitochondrial genes (bottom) across the same group. **D **The Venn diagram compares the number of non-variable genes removed using the computational method (SoupX, left) and the CRISPR-Cas9 method (right). The list of eliminated genes are shown in the accompanying boxes. **E** The UMAP shows the score of non-variable gene expression across different cell types in the original data (left) and CRISPR-Cas9-treated data (right). Red indicates high expression levels, while blue represents low expression levels. **F** The paired dot plots compare the median number of genes detected per cell (right) and the number of recovered cells (left) between the original dataset and CRISPR-Cas9-treated data. Cellranger (v7.0.1) was used for alignment. The *p*-value was calculated using a paired t-test. "ns" indicates no significant difference. scRNA-seq, single cell RNA sequencing; IBD, inflammatory bowel disease; GEX, gene expression; CRISPR, clustered regularly interspaced short palindromic repeats

We performed scRNA-seq on human intestine tissue from 17 inflammatory bowel disease (IBD) patients (Fig. 1A). During the gene expression (GEX) library construction process, we separated the libraries in two groups: one underwent selective removal of non-variable RNAs using CRISPR-Cas9, while the served as a control. To exclude batch effect, both groups used the same GEX libraries. After the library construction, untreated samples were sequenced at 50,000 read pairs per cell, while CRISPR-Cas9 treated samples were sequenced at half the depth (25,000 read pairs per cell). The number of sequencing cycles and the number of input cell were identical in both groups.

In intestinal scRNA-seq data, ribosomal and mitochondrial genes often exhibit high expression levels, so that these genes are identified as differentially expressed genes (DEG) (Supplementary Table S2). This can interfere with the analysis process, particularly when using DEG-based tools. Such issue may arise due to ambient RNAs caused by a high RNase environment or the inherently high endogenous expression of these genes. To address this problem, computational methods are typically applied to mitigate the impact of these non-variable RNAs on the data. We sought to investigate whether the CRISPR-Cas9-based method offers advantages over computational methods.

To assess the performance of CRISPR-Cas9 based method, we compared RNA expression levels of targeted non-variable RNAs such as ribosomal genes, mitochondrial genes across three datasets: the original (untreated) data, computationally processed data, and CRISPR-Cas9-treated samples (Fig. 1B, Supplementary Fig. S1). The original intestinal data exhibited high expression of almost ribosomal genes. Computationally processed data (SoupX) showed a general reduction in the expression of these genes. Although it could reduce expression levels in highly expressing cells, it was unable to eliminate them completely. In contrast, the CRISPR-Cas9 method almost entirely removed these genes. This difference is attributed CRISPR-Cas9 physically targeting cDNA, whereas computational method relies solely on theoretical calculations. Similar results were observed in mitochondrial genes (Fig. 1B). Violin plots provide a comprehensive view of this result (Fig. 1C). Next, we analyzed which genes were deleted by each method. Venn diagram illustrates that the CRISPR-Cas9 method effectively removed a broader range of non-variable genes (77 genes), whereas the computational method eliminated only 19 genes (Fig. 1D). While computational method can remove mitochondrial genes, but it was less effective in ribosomal genes. Additionally, the CRISPR-Cas9 method eliminated other non-variable genes that were not addressed by computational methods such as *EEF1A1 *and* MALAT1*. This result demonstrated that CRISPR-Cas9 effectively eliminated non-variable genes better than computational method, and this approach maybe useful in circumstances when these genes impede the findings. To confirm that non-variable genes were evenly expressed across samples, we calculated non-variable gene score. UMAP visualizations illustrated this score across each cell types (Fig. 1E). In the original data, non-variable RNAs were distributed quite evenly across all cell types. However, after CRISPR-Cas9 treatment, the non-variable gene score was decreased.

It was important to confirm that sequencing quality of both groups (original and CRISPR-Cas9 treated) was comparable. By eliminating non-variable RNAs, the CRISPR-Cas9 method reduced the total transcripts, allowing us to achieve similar data quality at lower sequencing depths. In this study, the original data were sequenced at 50,000 reads per cell, while the CRISPR-Cas9 data were sequenced at half that depth (25,000 reads per cell). Despite half of the sequencing depth, the CRISPR-Cas9 samples showed consistent sequencing saturation, median genes per cell, and recovered cell number (Fig. 1F, Table 2). This result indicate that the CRISPR-Cas9 method can produce high quality data at half the sequencing cost.

**Table 2:** Sequencing quality after alignment

	Saturation		Median genes per cell		Recovered cell	
Data	Original data	CRISPR-Cas9	Original data	CRISPR-Cas9	Original data	CRISPR-Cas9
GEM1	80.9%	74.6%	1366	1562	27,252	14,174
GEM2	70.8%	74.8%	1790	1865	16,724	16,467
GEM3	77.5%	84.0%	1110	1155	24,056	22,699
GEM4	73.6%	74.5%	1296	1342	22,149	20,677
GEM5	68.1%	68.2%	569	645	72,123	54,903
GEM6	70.0%	75.7%	1257	1403	23,867	22,135
GEM7	69.6%	74.3%	1363	1417	21,944	21,161
GEM8	55.2%	65.0%	1490	1530	21,040	20,538

### CRISPR-Cas9-based non-variable RNA deletion maintains consistent biological results

**Fig. 2:**
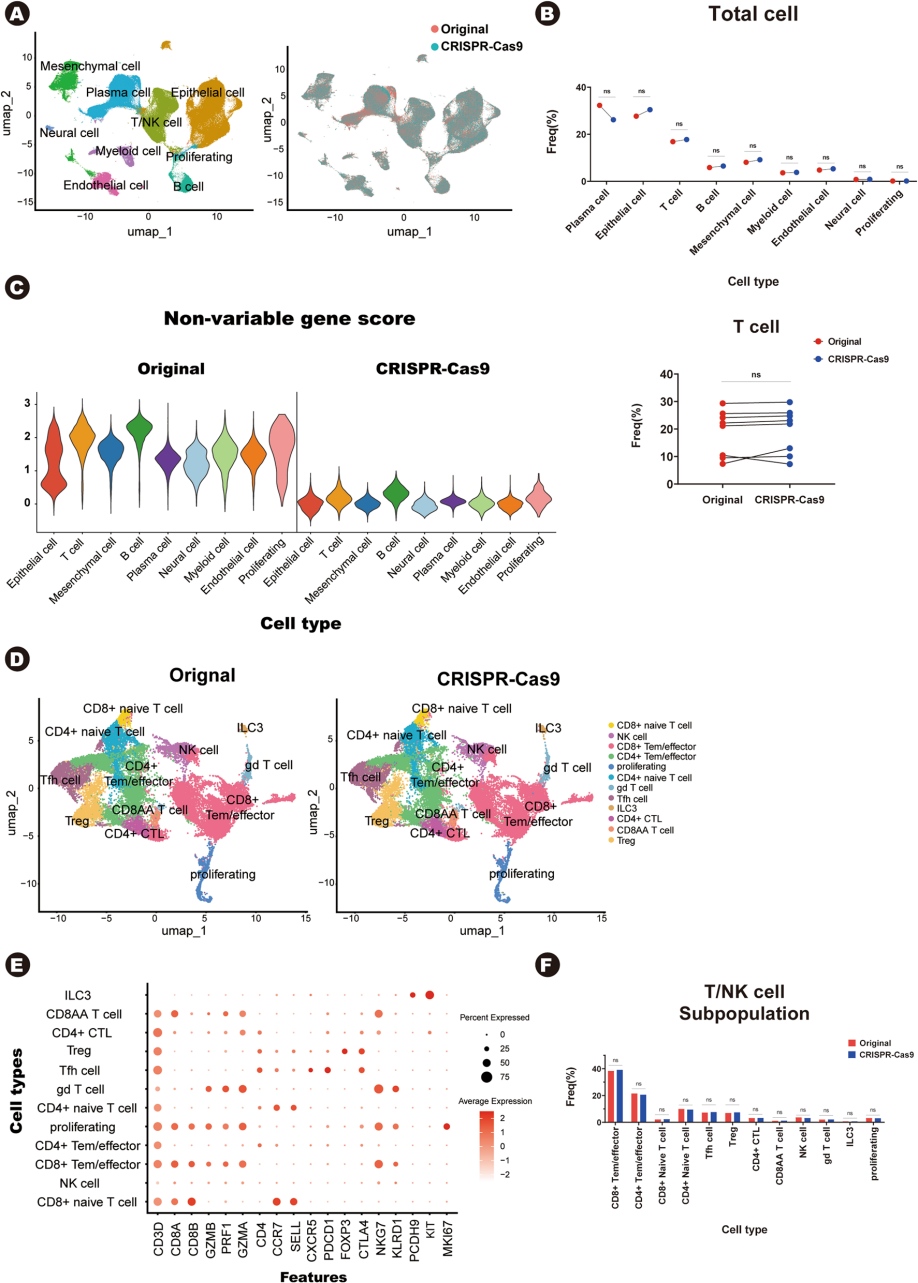
**A** UMAP visualization displaying 342,795 human intestinal cells from two groups (original and CRISPR-Cas9-treated), clustered into eight major cell types (left). The right panel shows UMAP visualization with cells colored by dataset. **B** The upper panel presents a paired dot plot showing the frequencies of eight major cell clusters. Red dots represent the original data and blue dots indicate CRISPR-Cas9-treated data. The bottom panel shows a paired dot plot showing the frequencies of T/NK cells across individual GEMs. Each dot represents a single GEM. “ns” indicates no significant difference. **C** Violin plots illustrating the score of non-variable gene expression across different cell types. **D** UMAP visualization showing 55,343 T/NK cells in two groups (left; original data, right; CRISPR-Cas9 data). **E** Dot plot showing the expression of marker genes in each T/NK cell subpopulation. **F** Bar plot comparing the frequencies of T/NK cell subpopulations between the two datasets. *P*-values were calculated using a two-tailed paired *t*-test

While we confirmed that CRISPR-Cas9 effectively deletes targeted non-variable gene, we were concerned that it might impact data integrity. So, we compared cell composition between the original data and the CRISPR-Cas9-treated data. Using the 10X Genomics Chromium platform, we obtained high-quality scRNA-seq data comprising 342,795 cells in total (183,061 cells from the original data and 159,734 cells from the CRISPR-Cas9-treated data). We annotated eight major cell clusters based on well- established marker genes [7], including plasma cells, epithelial cells, T cells, B cells, mesenchymal cells, myeloid cells, endothelial cells, and neural cells. The two groups integrated well, showing no batch effects (Fig. 2A, Supplementary Fig. 2 A–B). To compare cell type frequencies between the original and CRISPR-Cas9 treated data, we analyzed the distribution of each cell type (Fig. 2B, Supplementary Table S3). There were no significant differences in any of the cell types. Since Fig. 2B presents mean values, it may appear as there is a difference in cell frequency for plasma cells and epithelial cells. However, further examination of individual GEMs showed that there were no statistically significant differences in cell frequency (Fig. 2B,Supplementary Fig. S2 C). The observed variation in GEMs following CRISPR-Cas9 treatment may be due to quality control steps, such as doublet removal, which could have led to an unequal loss of cells between groups. Next, we sought to further demonstrate that CRISPR-Cas9 does not alter the data trends, even at the subpopulation level. In the original data, T/NK cells exhibited high levels of non-variable gene expression and represented the third largest cell population (Figs. 1E and 2B, C). So, we focused on T/NK cell and identified twelve T/NK cell subpopulations in both groups (original = 27,949 cells, CRISPR-Cas9 = 27,394 cells) (Fig. 2D, E). UMAP plots confirmed that the T/NK cell subpopulations integrated well (Supplementary Fig. S2D), and there were no significant differences in the T/NK cell subtype frequencies (Fig. 2F, Supplementary Table S4). We focused on T/NK cells, because plasma cells exhibited high heterogeneity, lacked a well-established subpopulation classification, and epithelial cells showed lower non-variable gene expression compared to T/NK cells (Fig. 2C). When we examined epithelial cell data, their frequencies also remained consistent across the original and CRISPR-Cas9-treated data (Supplementary Fig. S3 A–C, Supplementary Table S4). These results indicate that the CRISPR-Cas9 based method does not affect the integrity of scRNA-seq data, even after the removal of non-variable RNAs. And the CRISPR-Cas9 data retained biological information consistent with the original data.

### Validation of CRISPR-Cas9 efficacy and its impact on gene expression patterns

The CRISPR-Cas9 treated dataset showed sustained cellular composition. Although there were no significant differences in cell frequencies, the possibility remained that gene expression patterns of other genes could alter due to the change of non-variable genes. Additionally, we were concerned that CRISPR-Cas9 might mistakenly remove unintended genes which were not listed in guide RNA.

**Fig. 3:**
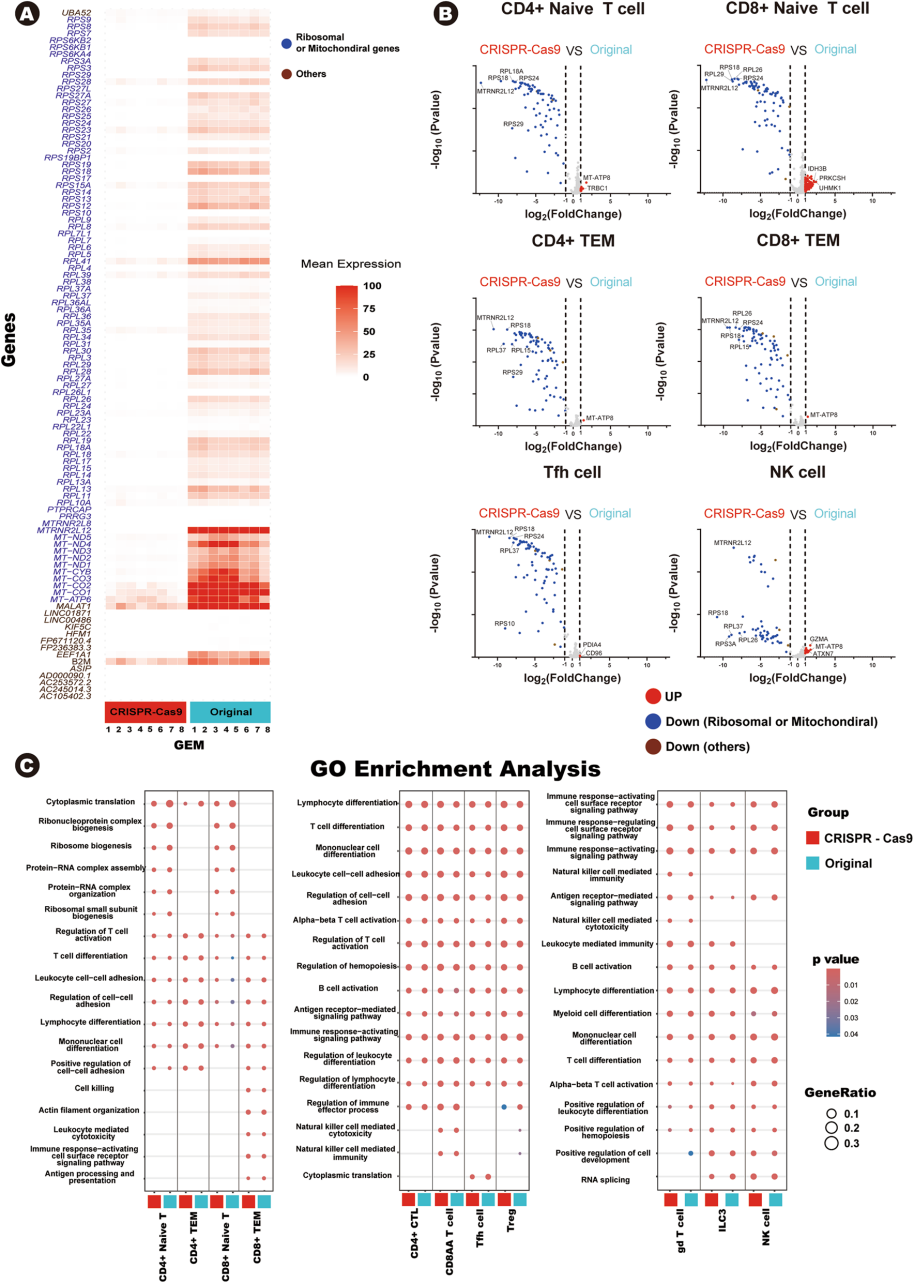
**A** Heatmap displaying downregulated genes in the CRISPR-Cas9 dataset. Color density indicates the expression levels of each gene. Among the genes, ribosomal genes and mitochondrial genes are highlighted in blue, while other genes are shown in brown.** B** Volcano plots illustrating differently expressed genes in original data compared to CRISPR-Cas9 data within six T/NK cell subpopulations. Upregulated genes in the original data are shown on the right, while downregulated genes are displayed on the left. Genes with *p*-value < 0.05 and log2 fold change > 1 or <− 1 are colored (right panel : log2 fold change > 1; left panel : log2 fold change <− 1). Among the downregulated genes, ribosomal genes and mitochondrial genes are highlighted in blue, while other genes are shown in brown.** (C)** Dot plots showing the top five GO pathways within T/NK subpopulation. Dot size represents the gene ratio participating in each pathway. *P*-values were calculated using a Wilcoxon rank-sum test. GO, gene ontology

To investigate this, we first compared the expression of all genes between the CRISPR-Cas9-treated and original data. The results indicated that have quite different expression between two groups. Among 32,056 genes, 101 genes were downregulated (log2 fold change < − 1) in the CRISPR-Cas9 treated data (Supplementary Table S5). Most of these genes were non-variable genes included in the guide RNA list, primarily ribosomal genes and mitochondrial genes, which is consistent with our result in Fig. 1. A heatmap showed genes that downregulated in CRISPR-Cas9 treated data (Fig. 3A). Expression levels in about 100 genes are decreased and most of genes are mitochondrial genes or ribosomal genes. Next, we investigated whether the same issue occurred within the subpopulation levels. Technically, the smallest nonzero *p*-value in R version 4.4 is 2.225074e − 308, and if the *p*-value falls below this threshold, it is displayed as 0. In our data, CRISPR-Cas9 almost entirely removed ribosomal genes and mitochondrial genes, leading to *p*-value of these genes being displayed as 0. This numerical limitation indicated complete removal but made it challenging to visualize the data. So, we randomly downsampled the dataset to retain biological information while increase *p*-value to manageable levels for R [8]. Volcano plots illustrated upregulated/downregulated genes in the original data compared to the CRISPR-Cas9 data within the T/NK cell subpopulation (Fig. 3B, Supplementary Fig. S4 A). Colored genes represent those with *p*-value < 0.05 and a log₂ fold change > 1 or < − 1, and genes highlighted in blue represent ribosomal or mitochondrial genes. Across all of T/NK cell subpopulations, no unintended gene deletions were detected. These findings demonstrate that the CRISPR-Cas9 method can effectively remove targeted genes without unintended data loss.

At last, we aim to demonstrate whether the removal of non-variable genes influenced analyses in two datasets. We conducted a pathway analysis using GO database (Fig. 3C). Each T/NK cell subpopulation of two groups (Original data and CRISPR-Cas9 treated data) inferred the same GO pathways. Since the inferred pathways remained consistent, we can carefully conclude that the CRISPR-Cas9 treated data remain consistent in gene expression analysis.

## Discussion

In some organs, high expression of RNase and DNase activity pose significant challenges in scRNA-seq experiments. These enzymes cause cell death during the isolation or GEM generation process, leading to an abundance of ambient RNA [9]. This ambient RNA inserts single-cell droplets, making it appear as if some cells inherently express certain RNAs. This problem is not neglectable, because it can result in misleading data, such as an overrepresentation of doublets or the appearance that the most cells uniformly express certain genes [10]. In intestinal scRNA-seq data, mitochondrial and ribosomal genes are often highly expressed. This overexpression can interfere with cell clustering and distort gene expression patterns. Therefore, for some organs prone to high ambient RNA contamination, alternative approaches are necessary, even if they come with some data loss. SoupX is commonly used to mitigate ambient RNA contamination by computationally identifying and removing genes inferred as ambient RNA. However, its specificity and efficiency are limited. However, based on our data, whether ribosomal and mitochondrial genes are increased due to ambient RNA from RNase activity or due to their inherently high endogenous expression, CRISPR-Cas9 provides a useful alternative for handling such data. In some studies, samples with high ribosomal and mitochondrial gene expression have been excluded to enhance analytical accuracy. However, the application of CRISPR-Cas9 allows for the retention of more data while minimizing bias.

In this study, from 352 genes (include in guide RNAs list), only 100 genes were successfully removed (Supplementary Fig. S4B). This indicates CRISPR-Cas9 we used (Jumpcode, DepleteX) had an efficiency of less than 30% compared to manufacturer data. The remaining 250 genes were either poorly removed or unaffected. This is likely due to the limitation in eliminating genes with naturally high expression or potential inefficiencies of guide RNA. However, mitochondrial and ribosomal genes were effectively removed, which was our primary objective, as these genes contribute to problems in intestinal tissue data analysis. Additionally, we expected that removing non-variable genes might reveal novel genes that were previously undetected in the original data because of their low expression levels. However, no such genes were identified in our dataset. A minor advantage was observed. Cell clusters that formed due to the high expression of mitochondrial and ribosomal genes disappeared. However, this was not a big deal, as these clusters were already part of other distinguishable clusters. Thus, we did not observe the expected improvements in cell clustering. From another perspective, if significant differences had emerged after CRISPR-Cas9 treatment, it would have been difficult to determine whether the whole results were reproducible in the original data. This may raise an confuse. However, fortunately, the CRISPR-Cas9-treated data remained consistent with the original results. This suggests that CRISPR-Cas9-treated data can serve as a alternative when the original dataset is noisy due to excessive non-variable gene expression or poor sample conditions.

In the field of scRNA-seq, small datasets or studies with limited cohort sizes often face challenges related to data reliability. As experiment scale and data size continue to grow, the sequencing burden also increases. From this perspective, our findings demonstrate that high-quality scRNA-seq data can be obtained at half the sequencing depth using the CRISPR-Cas9-based method. This means that high-quality data can be generated at half the cost, which is particularly beneficial for large-scale studies or datasets with high cell input Our data, which show comparable sequencing saturation and quality values, support this conclusion. Furthermore, this cost-efficient approach could be applied to other areas, such as bulk RNA sequencing and spatial transcriptomics, including technologies like 10X Visium (10X Genomics).

This study also has some limitations. The selected non-variable genes were assumed to have minor effects on data interpretation. As their name suggests, “non-variable genes” are ubiquitously expressed and are typically excluded from DEG based analyses. Moreover, these genes are generally not expected to play significant functional roles. However, in specific experimental contexts, their expression may offer valuable insights. And some databases or machine learning model may utilize data from these genes. Therefore, CRISPR-Cas9-based gene deletion should be selectively applied depending on the objectives of the experiment.

In Supplementary Figure S4, some NK/T cell subpopulations, such as ILC3 and CD8AA T cells, exhibited numerous downregulated genes beyond ribosomal and mitochondrial genes. We hypothesize that this phenomenon may result from the small number of cells in these populations, leading to potential statistical overestimation of certain genes.

Also, while computational methods often exhibit limited performance in removing ribosomal genes, they offer the advantage of eliminating other ambient RNAs within the data. By contrast, CRISPR-Cas9 can only target and remove genes that are specifically selected using guide RNA. It is important to note that we do not claim that the performance of SoupX in intestinal scRNA-seq data is inadequate; rather, we compare their efficacy specifically in the context of certain genes.

In conclusion, CRISPR-Cas9-based non-variable gene deletion can be particularly useful when studying organs with high expression of non-variable genes or when working with large datasets. However, this approach may not be suitable for datasets where these genes provide valuable information. In future studies, we will compare our data with results from other CRISPR-Cas9 methods using different guide RNAs. This comparison will help determine whether the observed trends are consistent across various CRISPR-Cas9-based methods.

The cohort data are provided in Supplementary Table S6.

## Supplementary Information

Supplementary Material 1: Supplementary Figure S1. The feature plots from UMAP visualization show the expression levels of ribosomal and mitochondrial genes across three groups: original data (left), computationally processed data using SoupX (in silico) (middle), and CRISPR-Cas9 treated data (right).

Supplementary Material 2: Supplementary Figure S2. (A) Dot plot showing the expression of marker genes in intestinal tissue data. (B) UMAP visualization of total cells from each two group : original (left) and CRISPR-Cas9-treated data (right), clustered into nine major cell types. (C) Paired dot plots showing the frequencies of each cell type across individual GEMs. Red dots represent the original data, while blue dots indicate CRISPR-Cas9-treated data. Each dot represents a single GEM. “ns” indicates no significant difference. (D) UMAP visualization of NK/T cells from two groups (original and CRISPR-Cas9-treated) with cells colored by dataset. *P*-values were calculated using a two-tailed paired t-test.

Supplementary Material 3: Supplementary Figure S3. (A) UMAP visualization of intestinal epithelial cells from two groups (original and CRISPR-Cas9-treated), clustered into nine major cell types (top panel). The bottom panel shows UMAP visualization with cells colored by dataset. (B) Bar plot comparing the frequencies of epithelial cells in each individual GEM between the original and CRISPR-Cas9 treated data. (C) Bar plot comparing the frequencies of epithelial cell subpopulations between the two datasets. "ns" indicates no significant difference. *P*-values were calculated using a two-tailed paired t-test.

Supplementary Material 4: Supplementary Figure S4. (A) Volcano plots illustrating differently expressed genes in original data compared to CRISPR-Cas9 data within five T/NK cell subpopulations. Upregulated genes in the original data are shown on the right, while downregulated genes are displayed on the left. Genes with *p*-value <0.05 and log2 fold change>1 or < -1 are colored (right panel : log2 fold change >1; left panel: log2 fold change < -1). Among the downregulated genes, ribosomal genes and mitochondrial genes are highlighted in blue, while other genes are shown in brown. (B) Bar plot displaying 352 genes from the CRISPR-Cas9 guide RNA list. Removed genes (log2 fold change < 0) are shown as blue bars, while non-removed genes (log2 fold change > 0) are shown as red bars. The names of ribosomal genes and mitochondrial genes are highlighted in blue.

Supplementary Material 5: Supplementary Table S1. The list of guide RNAs.

Supplementary Material 6: Supplementary Table S2. Differentially expressed genes of epithelial cell in original data.

Supplementary Material 7: Supplementary Table S3. The frequencies of each cell type in original data and CRISPR-cas9 treated data.

Supplementary Material 8: Supplementary Table S4.The frequencies of the T/NK cell subtypes and epithelial cell subtypes.

Supplementary Material 9: Supplementary Table S5. The DEG between the CRISPR-Cas9-treated and original data.

Supplementary Material 10: Supplementary Table S6. The cohort data.

## Data Availability

.The data supporting this study are not publicly available. Any requests for data should be directed to the corresponding author and will be considered based on institutional and ethical guidelines
