# Supplementary figures and images for: Non-variable RNA deletion using the CRISPR-Cas9 technique demonstrated improved outcomes in human intestine single-cell RNA sequencing data, even at half sequencing depths
Source: Genomics Inform. 2025 May 20;23:14. doi: 10.1186/s44342-025-00043-6 (PMC12093678; doi:10.1186/s44342-025-00043-6)

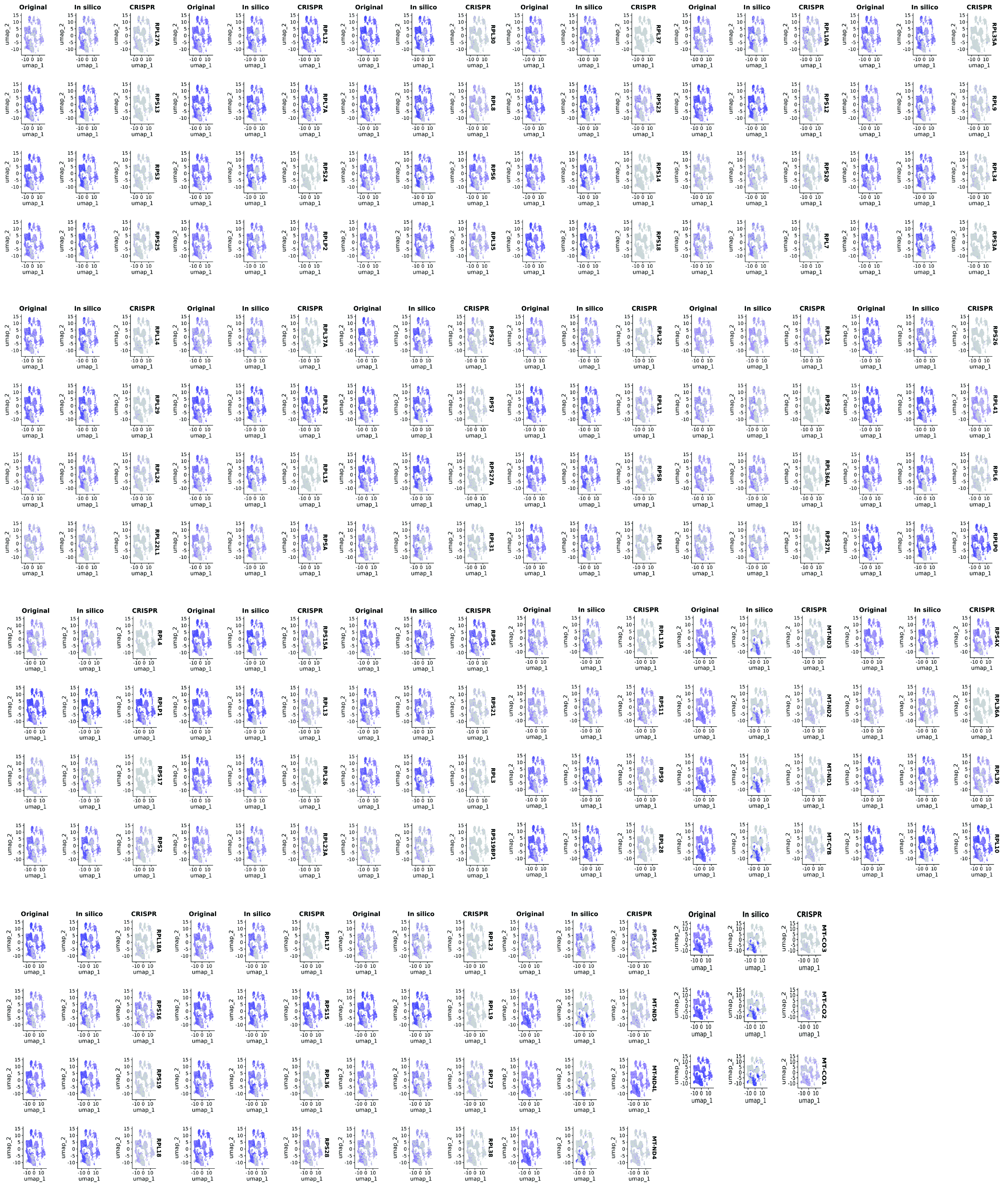

Supplement: Supplementary file 1 — Supplementary Material 1: Supplementary Figure S1. The feature plots from UMAP visualization show the expression levels of ribosomal and mitochondrial genes across three groups: original data (left), computationally processed data using SoupX (in silico) (middle), and CRISPR-Cas9 treated data (right). [file 44342_2025_43_MOESM1_ESM.tif]

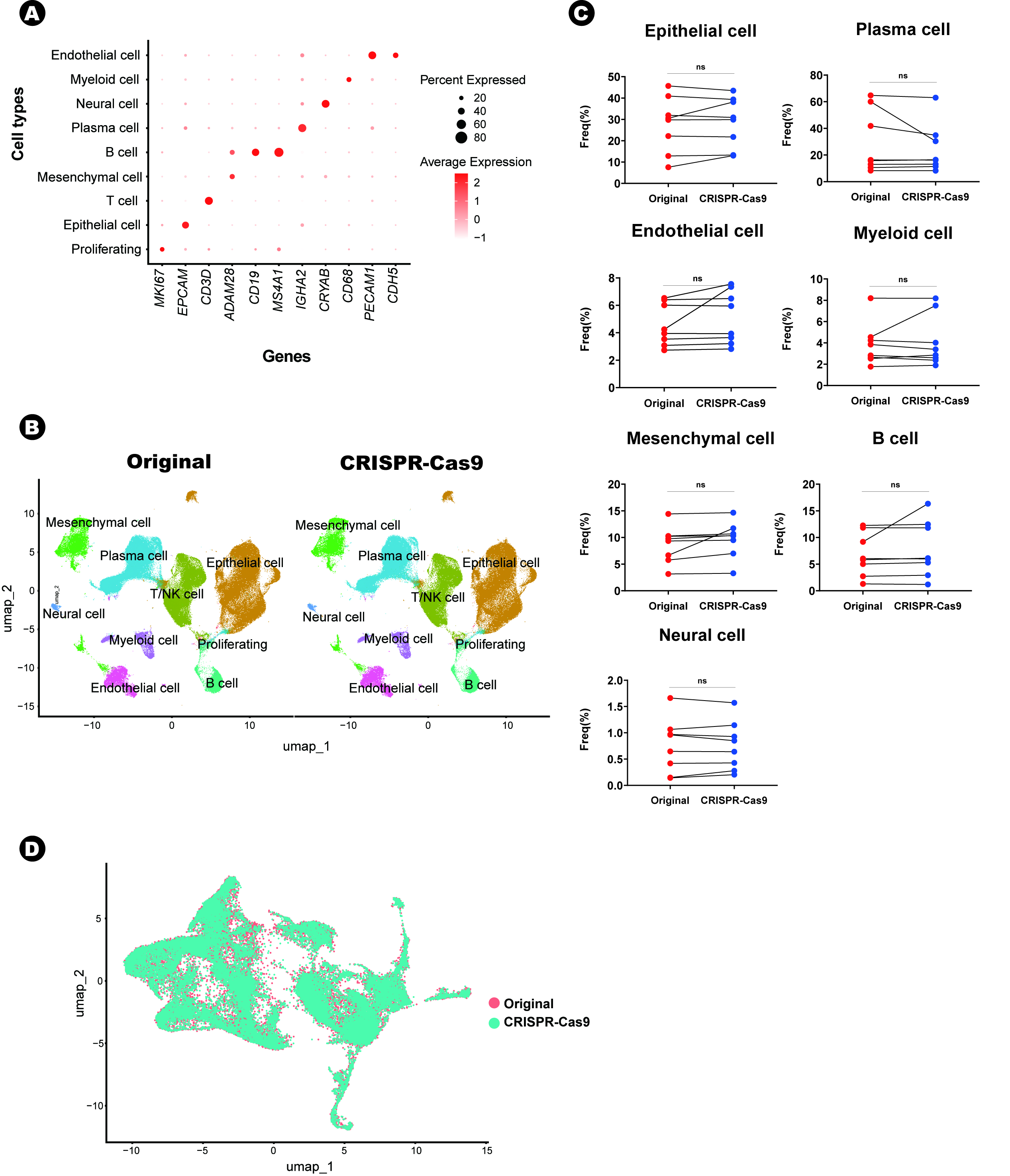

Supplement: Supplementary file 2 — Supplementary Material 2: Supplementary Figure S2. (A) Dot plot showing the expression of marker genes in intestinal tissue data. (B) UMAP visualization of total cells from each two group : original (left) and CRISPR-Cas9-treated data (right), clustered into nine major cell types. (C) Paired dot plots showing the frequencies of each cell type across individual GEMs. Red dots represent the original data, while blue dots indicate CRISPR-Cas9-treated data. Each dot represents a single GEM. “ns” indicates no significant difference. (D) UMAP visualization of NK/T cells from two groups (original and CRISPR-Cas9-treated) with cells colored by dataset. P-values were calculated using a two-tailed paired t-test. [file 44342_2025_43_MOESM2_ESM.tif]

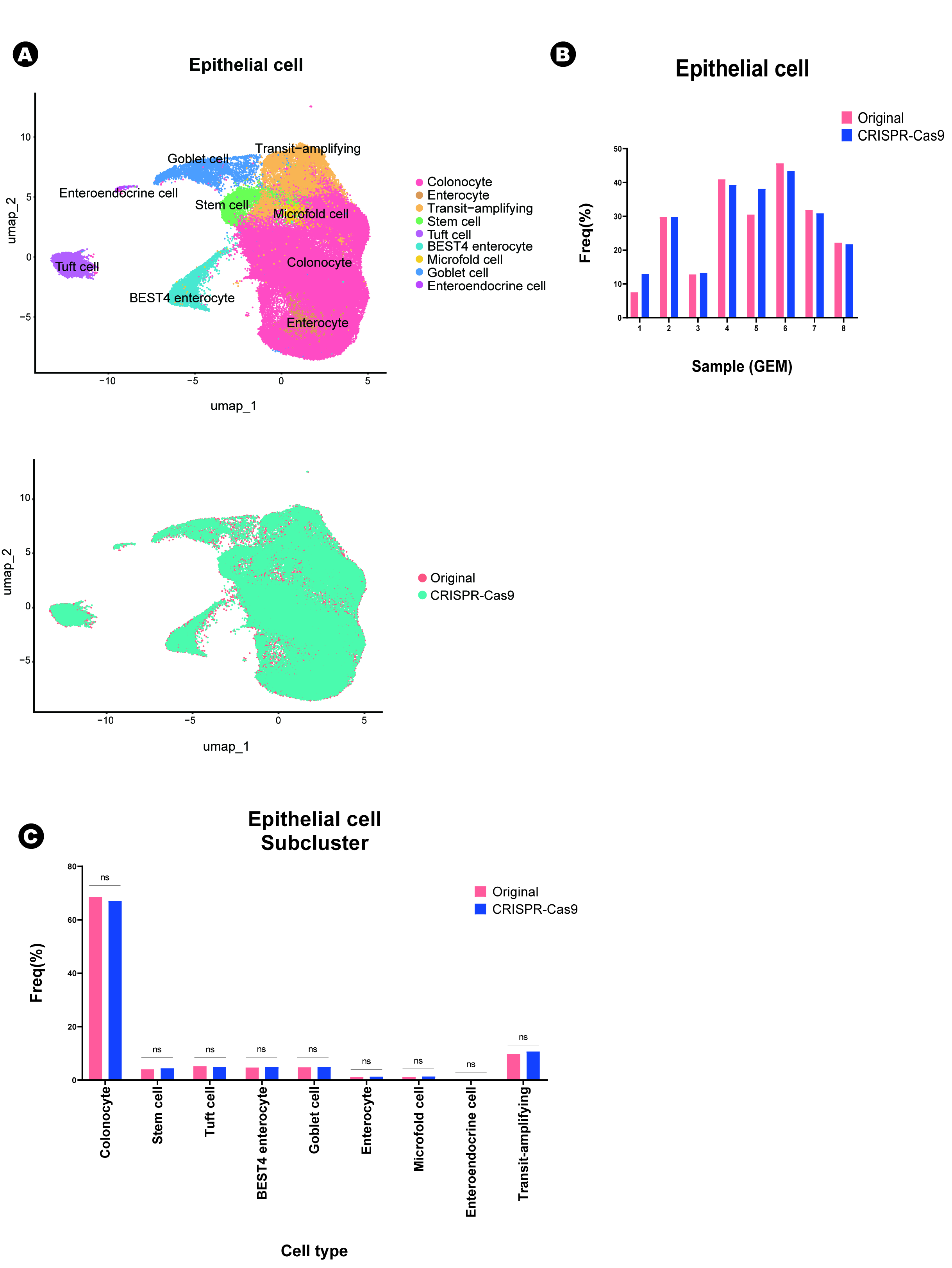

Supplement: Supplementary file 3 — Supplementary Material 3: Supplementary Figure S3. (A) UMAP visualization of intestinal epithelial cells from two groups (original and CRISPR-Cas9-treated), clustered into nine major cell types (top panel). The bottom panel shows UMAP visualization with cells colored by dataset. (B) Bar plot comparing the frequencies of epithelial cells in each individual GEM between the original and CRISPR-Cas9 treated data. (C) Bar plot comparing the frequencies of epithelial cell subpopulations between the two datasets. "ns" indicates no significant difference. P-values were calculated using a two-tailed paired t-test. [file 44342_2025_43_MOESM3_ESM.tif]

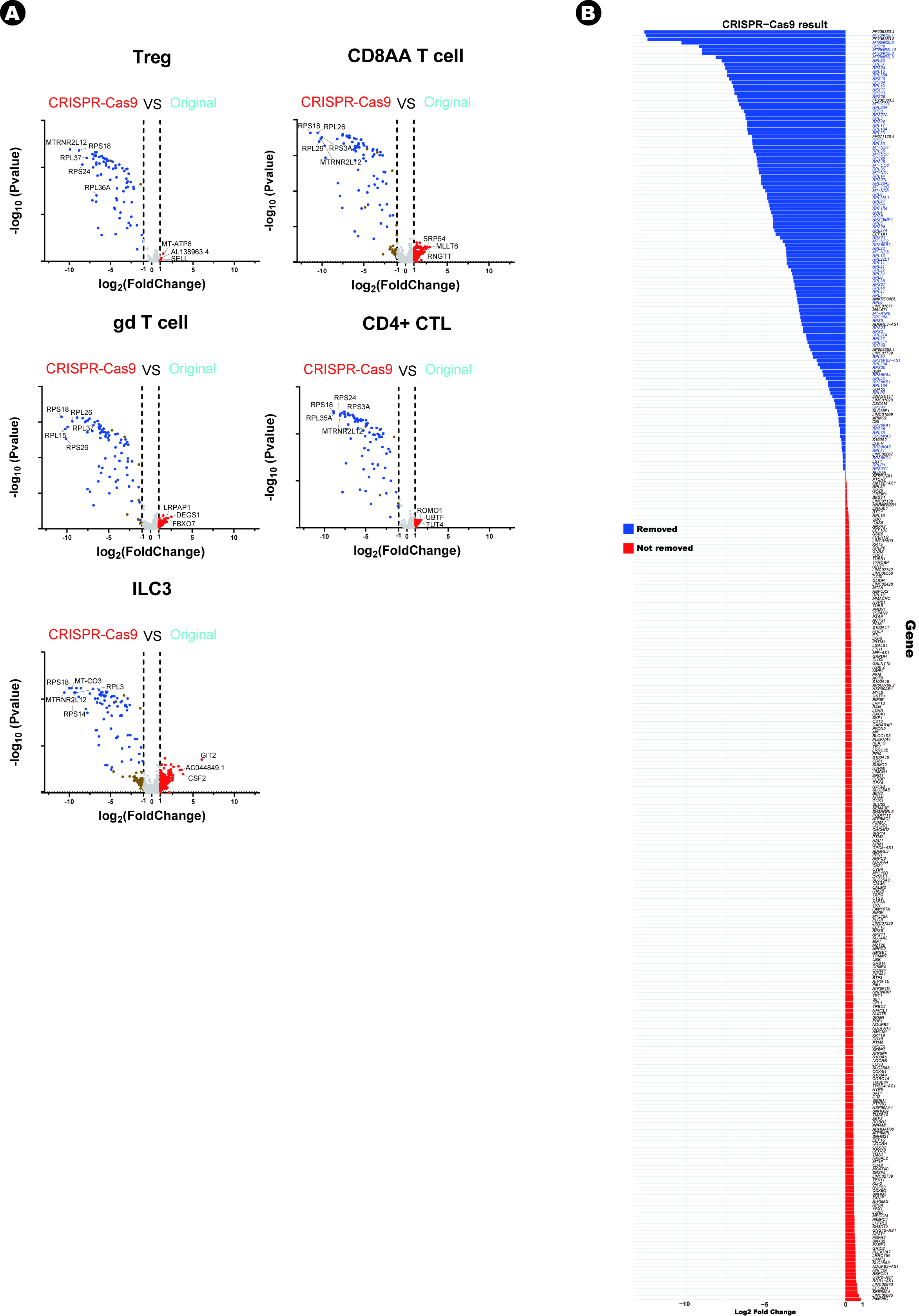

Supplement: Supplementary file 4 — Supplementary Material 4: Supplementary Figure S4. (A) Volcano plots illustrating differently expressed genes in original data compared to CRISPR-Cas9 data within five T/NK cell subpopulations. Upregulated genes in the original data are shown on the right, while downregulated genes are displayed on the left. Genes with p-value <0.05 and log2 fold change>1 or < -1 are colored (right panel : log2 fold change >1; left panel: log2 fold change < -1). Among the downregulated genes, ribosomal genes and mitochondrial genes are highlighted in blue, while other genes are shown in brown. (B) Bar plot displaying 352 genes from the CRISPR-Cas9 guide RNA list. Removed genes (log2 fold change < 0) are shown as blue bars, while non-removed genes (log2 fold change > 0) are shown as red bars. The names of ribosomal genes and mitochondrial genes are highlighted in blue. [file 44342_2025_43_MOESM4_ESM.tif]
